# Consequences of contact restrictions for long-term care residents during the first months of COVID-19 pandemic: a scoping review

**DOI:** 10.1007/s10433-023-00787-6

**Published:** 2023-10-17

**Authors:** Petra Benzinger, Hans-Werner Wahl, Jürgen M. Bauer, Anne Keilhauer, Ilona Dutzi, Simone Maier, Natalie Hölzer, Wilco P. Achterberg, Natascha-Elisabeth Denninger

**Affiliations:** 1 Center for Geriatric Medicine, Heidelberg University Hospital, AGAPLESION Bethanien Krankenhaus Heidelberg, Rohrbacher Strasse 149, 69126 Heidelberg, Germany; 2 Institute of Health and Generations, University of Applied Sciences Kempten, Bahnhofstrasse 61, 87435 Kempten, Germany; 3 Network Aging Research (NAR), Heidelberg University, Bergheimer Str. 20, 69115 Heidelberg, Germany; 4 Department of Public Health and Primary Care, Leiden University Medical Center, Leiden, The Netherlands; 5 Centre for Research, Development and Technology Transfer, Technical University of Applied Sciences Rosenheim, Hochschulstraße 1, 83024 Rosenheim, Germany; 6 Medical Faculty, Institute for Health and Nursing Science, International Graduate Academy, Martin-Luther-University Halle-Wittenberg, Magdeburger Straße 8, 06112 Halle (Saale), Germany; 7 Medical Faculty Heidelberg, Department of General Practice and Health Services Research, Nursing Science and Interprofessional Care, Heidelberg University, Im Neuenheimer Feld 130.3, 69120 Heidelberg, Germany

**Keywords:** Coronavirus, Health consequences, Quality of life, Nursing home, Care facility, Loneliness

## Abstract

During the early stages of the COVID-19 pandemic, stringent measures were implemented in most countries to limit social contact between residents of long-term care facilities (LTCF) and visitors. The objective of this scoping review was to identify and map evidence of direct and indirect consequences of contact restrictions, guided by three conceptual perspectives: (1) stress and learned helplessness (i.e., failure to use coping behaviors even when they are available and actionalble); (2) social contact loss; and (3) ‘total institution’ (i.e., a facility operates following a fixed plan due to spelled-out rules and norms, controlled by institutional representatives). We used the framework for conducting a scoping review by Arksey and O'Malley; included were peer-reviewed manuscripts reporting on the outcomes of contact restrictions from the beginning of the pandemic until the end of 2020. After removing duplicates, 6,656 records were screened and 62 manuscripts included. Results pertaining to the stress and learned helplessness perspective primarily focused on depressive symptoms, showing substantial increases compared to the pre-pandemic period. Studies examining cognitive and functional decline, as well as non-COVID-19 related mortality, were limited in number and presented mixed findings. The majority of study outcomes related to the social contact loss perspective focused on loneliness, but the study designs did not adequately allow for comparisons with the pre-pandemic status. The evidence concerning outcomes related to the 'total Institution' perspective was inconclusive. Although detrimental effects of social isolation in the long-term care context found support particularly in the negative affect domain, other outcome areas did not allow for definitive conclusions due to considerable variations in findings and, in some cases, insufficient statistical power.

**Supplementary Information:**

The online version contains supplementary material available at 10.1007/s10433-023-00787-6.

## Background

In January 2020, the first cases of what is now known as COVID-19 disease were reported internationally. The vulnerability of residents in long-term care facilities (LTCF) was starkly illustrated by an initial COVID-19 outbreak in Washington State. In this particular LTCF, 26% of the residents who were infected with the virus succumbed to the disease, marking a highly impactful event (McMichael et al. 2020). Shortly afterward, similar events occurred in countries such as Spain and Italy. The image captured by Emanuele di Terlizzi on the evening of March 18, 2020, depicting a procession of military trucks carrying predominantly older institutionalized individuals in the city of Bergamo, northern Italy, has left an indelible mark at least on the collective European memory of the pandemic.

In response to the growing number of outbreaks in LTCFs, the World Health Organization issued a recommendation on visitor restrictions in March 2020. Governments wordwide imposed visitation bans and most LTCFs stopped personal visits from family members and friends. In a parallel measure, social and support services provided by volunteers and external health services were banned in most LTCFs. In addition, to prevent transmission between asymptomatically infected residents, social interactions among residents were drastically reduced by suspending communal meals and leisure activities.

Although framed by most experts as unavoidable, concerns about the negative consequences of these rather drastic social isolation measures for large portions of the long-term care resident population have been raised from the beginning of the pandemic (Abbasi 2020). Although various reviews have synthesized the physical and mental health consequences of the COVID-19 pandemic in the general population at different stages of the pandemic (Clemente-Suárez et al. 2020; Prati and Mancini 2021; Vindegaard and Benros 2020), only one review (Lebrasseur et al. 2021) considered to the best of our knowledge in their study pool of 135 paper a small portion of three studies addressing LTCF residents, rendering it insufficient to extract specific findings for this particular subpopulation (Lebrasseur et al. 2021). Benzinger et al. (2021) published a systematic synthesis of 15 early studies (data collection between February and June 2020) that rather consistently found negative outcomes for residents, proxies and health care professionals (HCP). However, due to the limited time period considered in this review, extension of the data basis is needed.

### Living in LTCFs under COVID-19 constraints: conceptual perspectives

We argue that the empirical examination of the physical and mental health consequences of contact restrictions in LTCF settings during the COVID-19 pandemic requires sufficient conceptual underpinning, an issue that has been largely ignored in the previous pandemic literature to date. Therefore, we propose theoretical perspectives with high relevance for the living situation of older adults in LTCFs and their exposure to the constraints of the COVID-19 pandemic, in particular: (1) the critical life event, stress theory and learned helplessness conceptual perspective; (2) the social contact loss conceptual perspective; and (3) the ‘total institution’ conceptual perspective.

The critical life event and stress theoretical approach (Aldwin et al. 2021) suggests that anticipation of and perceived control over the critical life events helps to reduce the experienced stress burden of the events. In the social sphere, anticipating social isolation and disruption of one’s social world and experiencing at least some control over the impending social life event would be beneficial (Carton and Aiello 2009). Translated to the pandemic and the containment strategies implemented, the unpredictable nature of the COVID-19 outbreak, its consequences in terms of unexpected quarantining and social isolation, and the lack of previous experience with a similar event, residents had a very limited repertoire of coping strategies with proven success at their disposal. Indeed, a state of ‘learned helplessness', characterized by a failure to use coping behaviors even when they are available and actionalble (Duru and Balkıs, 2022; Seligman 1975), may have occurred as a result of the abrupt and strict social isolation with negative consequences as described in the helplessness theory literature (Duru and Balkıs, 2022; Seligman 1975). That is, detrimental effects in the domains of socio-emotional, cognitive-executive, and physical functioning, as well as in all-cause mortality.

Secondly, in terms of the social contact loss conceptual perspective, the isolation of residents in their rooms, coupled with the absence of visitors, was arguably the most critical and detrimental consequence of managing the pandemic in the LTCFs. Self-determination theory (Ryan and Deci 2017), as well as other conceptualizations (Baumeister and Leary 1995) posit that ‘connectedness’ and the feelings of belonging are paramount to human development. Translated to the pandemic and its subsequent protective measures, visits from family and friends served to maintain perceptions of continuity, appreciation, and ties with the outside world (Baumeister and Leary 1995; Kang et al. 2020). Support from relatives and friends is also important, if not critical, for communicating potentially unmet needs and experienced problems in LTCF daily routines (Kang et al. 2020). In fact, many relatives not only remain emotionally attached to their loved ones who have moved to care facilities, but often spend many hours a week in the facilities and are actively involved in their care (Whitlatch et al. 2001). Therefore, the social contact loss conceptual perspective suggests negative outcomes in terms of loneliness, decreased overall quality of life, and eventually also in areas such as loss of appetite and body weight.

Thirdly, Goffman’s (1961) conceptual perspective of ‘total institutions’ deserves consideration. ‘Total institutions’ are typically concentrated in one place and have a centrally installed authority. Activities of those living in 'total institutions' underlie a fixed plan and their flow happens due to spelled-out rules and norms that are controlled by institutional representatives. All such plans and activities are monitored and optimized in order to attain the major goals of the institution neglecting to a large extent individual differences. Caution should certainly be expressed in generalizing the characteristics of a ‘total institution’ in Goffman’s sense to LTCFs (Clark and Bowling 1990). However, applied to the pandemic and the containment strategies implemented, there is reason to believe that what happened in many LTCFs during the pandemic brought them closer to the concept of a ‘total institution’ and its consequences on those living within such facilities (Ayalon and Avidor 2021). One characteristic of ‘total institutions’ refers to the necessity for much increased internal control measures and banning most activities allowing for social interaction neglecting individual-level differences and preferences for the sake of keeping the institution ‘safe'. As a consequence, those living in ‘total institutions’ experience a far-reaching loss of autonomy. Typical indicators of such increased ‘totality', may include changes in LTCFs such as increases in neuropsychiatric symptoms, the use of psychoactive medications, and eventually physical restraint use.

## Research goals and expectations

The aim of this review is to provide a conceptually driven synthesis of the available evidence on the physical and mental health consequences of contact restriction and various form of social isolation as a potentially multifaceted response to the challenges of the COVID-19 pandemic for older adults living in LTCFs. We focused on the period after the implementation of contact restrictions, specifically from the beginning of these restrictions until the end of 2020. Based on relevant theoretical frameworks, our overarching hypothesis is that the social isolation experienced by LTCF residents during the COVID-19 pandemic resulted in adverse outcomes across multiple domains. Based on the three conceptual lines as outlined above, we arrived at the following expectations:

1. Informed by the stress and learned helplessness conceptual perspective, we expected to see adverse outcomes such as increased depression, lowered cognitive and functional status, and heightened non-COVID-19 related mortality;
2. Informed by the social contact loss conceptual perspective, we expected to see adverse outcomes such as increased loneliness, appetite loss;
3. Informed by the ‘total institution’ conceptual perspective, we expected to see adverse outcomes such as increased neuropsychiatric symptoms, prescription of psychoactive drugs.

## Methods

This scoping review followed the framework proposed by Arksey and O'Malley (Arksey and O’Malley 2005). The results are reported in accordance with PRISMA Extension for Scoping Reviews (Tricco et al. 2018) (Additional file 1: Table S1). A protocol was published (see https://osf.io/g2yav/?view_only=38f0c6caebec4afb90378dc6726fc1ea).

### Identifying potentially relevant studies

An initial search of MEDLINE was performed via PubMed, driven by the three domains of outcomes suggested by our conceptual background, and a limited number of retrieved articles were screened to develop a final search strategy with the goal to maximize sensitivity (Additional file 1: Table S3). This strategy was translated to all other databases: MEDLINE via PubMed (including PubMed Central (PMC), in-process and other non-indexed citations, Epub-ahead-of-print articles, and author manuscripts), EMBASE, CINAHL via EBSCOhost, PsycINFO via EBSCOhost, Web of Science (including Science Citation Index-EXPANDED), AgeLine (until 06/2021 only), and Cochrane Library. Initially, we searched all databases from January 2020 to May 2021 and limited our search to publications in English, German, and Dutch. The search was updated in November 2022. We checked reference lists of reports identified for additional potentially eligible trials or ancillary publications. For removal of duplicates, screening, and further reviewing, search results were imported into the Covidence systematic review software from Veritas Health Innovation, Melbourne, Australia. Pre-prints, protocols and conference abstracts were checked for possible subsequent publication while only full manuscripts published in peer-reviewed journals were considered for further analyses.

### Study selection and inclusion criteria

Screening and further reviewing was executed by two researchers each. Based on the inclusion criteria, they screened title and abstract, and then any potentially relevant full texts. Any disagreements were resolved through discussion.

For studies to be included, they had to fulfill the following criteria:

- *Participants* This scoping review was restricted to people aged ≥ 60 years living in a LTCF without SARS-CoV-2 infection. Studies were included where results were stratified by infection status. For the definition of LTCF we accepted all non-acute residential and nursing facilities that housed exclusively older adults and serve them with a specified and enduring form of care that may be needed at any time of day.
- *Context* During the first months of the COVID-19 pandemic, wide-reaching contact restrictions for care facilities were implemented in most places in order to avoid spreading of the virus from the wider community to those most vulnerable. Restrictions ranged from reducing the number of visitors to limiting access to care facilities for those being employed in direct care. The extent of restrictions varied during the pandemic and by December 2020 the availability of vaccines rapidly changed management of contact restrictions in many places. Hence, this review only included studies reporting on consequences observed until the end of 2020.
- *Concept* The purpose of this review was to identify social, psychological, psychiatric, and other health consequences attributed to contact restrictions.

### Charting the data

A standardized chart form was developed by the reviewers to collect data. Two of the authors independently extracted the data from each full manuscript included in this review with the corresponding author synthesizing the data extractions. Study quality was not assessed, as the primary purpose of our scoping review was to map existing research activity.

### Collating, summarizing, and reporting the results

Findings of this scoping review are presented in a tabular format (see Additional file 1). A descriptive narrative summary presents the key issues thematically.

## Results

**Fig. 1:**
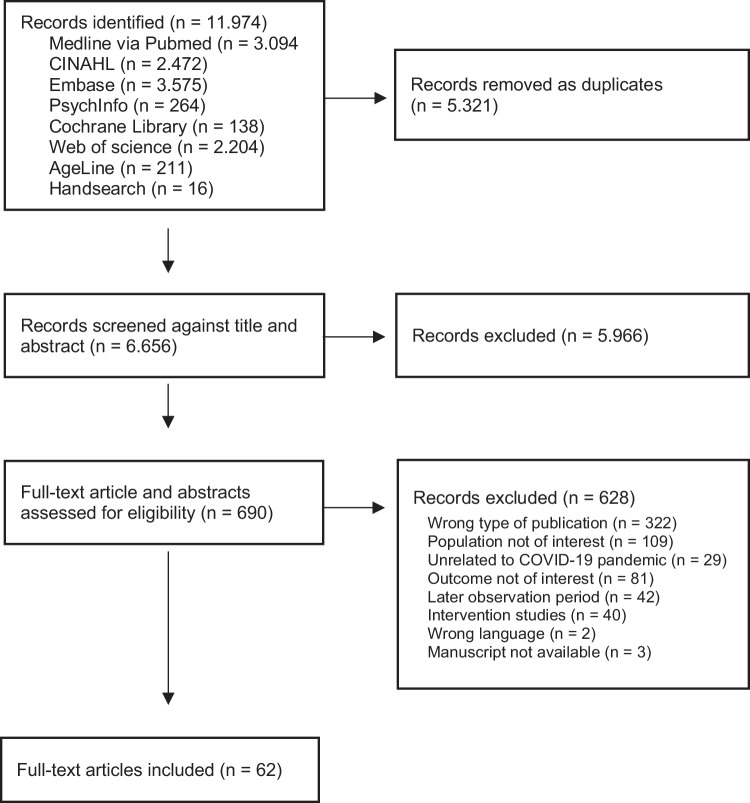
PRISMA flow chart

Our search in seven databases and hand search of key journals yielded 6,656 abstracts after removing duplicates. The flowchart of the search and selection process is shown in Fig. 1.

After full-text screening, 62 full texts were included in the narrative synthesis. The characteristics of the included studies are shown in Table 1.

**Table 1:** Characteristics of included studies

Author (first)	Study period		Source of data	Outcome parameter(s) of interest
	Pandemic	Pre-pandemic		
Akhtar-Danesh et al. (2022)	03/20–12/20	01/19–12/19	RAI-MDS, laboratory results	Mortality rates
Ali et al. (2022)	01/20–12/21	01/19–12/19	National Prescription Audit database	Prescription of antipsychotic drugs
Angevaare et al. (2022)	03/20–05/20	03/17–03/20	interRAI data transferred to research database	Self-reported mood, observer-rated mood, withdrawal, aggressive behavior, sleep, body weight, delirium
Arpacıoğlu et al. (2021)	12/20		Telephone interviews	Turkish death anxiety scale, Satisfaction with life scale, Depression, anxiety and stress scale-21 (DASS-21)
Barnett et al. (2022)	01/20–11/20	01/18–11/18 and 01/19–11/19	MDS, Medicare claims data	Mortality, ADLs, body weight, PHQ-9
Campitelli et al. (2021)	03/01/20–09/26//20	2017–2019	Administrative data and Ontario Drug Benefit database	Prescription of psychotropics, anticonvulsants, opioids, antibiotics, antihypertensive medication
Chang et al. (2021)	09/20		Assessment by research personel	Self-rated health, leisure social support, flow, loneliness
Cheung et al. (2022)	03/20–06/20	03/19–06/19	interRAI	Self-rated health, ALDs, Cognitive Performance Scale, Depression Rating Scale, hospitalization
Cornally et al. (2022)	06/20		Online-survey	Self-perceived health
Cortés Zamora et al. (2022)	03/20–11/20		Health records and assessment by on-site caregiver	Barthel Index, Functional Ambulation Classification
Danilovich et al. (2020)	04/20	12/19 -03/20	Care plan	Body Weight
Davies-Abbott et al. (2021)	07/20		Interviews	Isolation, resilience, well-being and threat
Egeljić-Mihailović et al. (2022)	03/20–04/20		Self-assessment	GDS-15, Maastricht Social Participation Profile (MSPP)
El Haj et al. (2020)	04/20		Assessments by on-site caregiver	HADS
El Haj et al. (2020a)	03/20–11/20		Rating by on-site caregiver	Hallucination score (generic six-item questionnaire)
El Haj et al. (2021a, b)	05/20–11/20		Assessments by on-site caregiver	Depression (single-item question)
El Haj et al. (2022)	03/20–11/20		Assessments by on-site caregiver	HADS and 1-item question on loneliness
El Haj and Gallouj 2022)	n.a		Assessments by on-site caregiver	HADS and 1-item question on loneliness
Gerlach et al. (2021)	03/20–06/20	01/19–02/20	MDS	Prescription of psychoactive medication
Giebel et al. (2022)	05/20–11/20		Interviews	Well-being and health
Górski et al. (2022a, b)	03/20–12/20		Assessment by on-site caregiver	Risk of depressive symptoms (generic instrument)
Górski, Garbicz, et al. (2022)	02/20–05/20		Assessments by on-site caregiver	GDS-15
Greco et al. (2021)	06/20–07/20	10/19–12/19	Case–control study within ongoing study	Handgrip strength, walking speed, Mini-Mental State Examination, Frail-NH scale
Gustafsson, Fonseca-Rodríguez, et al. (2022)	03/20–05/20	03/19–05/19	Annual survey	Loneliness, self-rated health, worries and anxiety, indoor mobility limitations
Gustafsson, Schröders, et al. (2022)	02/20–05/20	02/19–05/19	Annual survey	Loneliness, self-rated health, worries and anxiety, indoor mobility limitations
Hindmarch et al. (2021)	06/20–09/20		Online survey, follow-up focus groups	Cognition and behavior
Ho et al. (2022)	06/20–07/20		Interviews	Loneliness
Hovey and Shropshire (2021)	04/20–05/20		Interviews	Well-being
Hua and Thomas (2021)	06/20–01/21		National Health and Aging Trends Study	Loneliness
Ickert et al. (2021)	07/20–10/20		Interviews	Well-being
Jones et al. (2022)	03/20–12/20	03/18–12/18 and 03/19–12/19	Administrative data, hospital information systems	Characteristics, in-hospital care, and outcomes for admissions to general medicine wards for non-COVID-19 reasons
Kaelen et al. (2021)	06/20		Focus group interviews	Well-being
Kiyoshi-Teo et al. (2022)	05/20		Interviews and paper–pencil surveys	Perception of fall risks, worry about falling, well-being
Koopmans et al. (2022)	05/20		Online survey and telephone interviews	Well-being
Leontjevas et al. (2021)	04/20–07/20		Online survey and interviews	Challenging behavior
Levere et al. (2021)	03/20–07/20		Minimum Data Set	Depressive symptoms, body weight, pressure ulcer, incontinence, Cognitive Functioning Scale
Li et al. (2022)	06/20–09/20		Administrative data	Non-COVID-19 related deaths
Lombardo et al. (2020)	03/20–04/20		Online survey	Use of physical restraint measures, use of psychoactive drugs, adverse events
Martinchek et al. (2021)	03/20–05/20		Medical records	Body weight
McArthur et al. (2021)	03/20–06/20	01/17–03/20	interRai	Depression, delirium, behavioral problems
Nair et al. (2021)	06/20–08/20		n.a	GDS- 30, Beck’s Anxiety Inventory, Multidimensional Scale of Perceived Social Support
Nash et al. (2021)	09/20–10/20		Online survey	Well-being
Paananen et al. (2021)	05/20–12/20		Face-to-face interviews	Well-being
Pereiro et al. (2021)	07/20–09/20	12/18–03/20	Health records	GDS-15, Barthel Index, Clinical Dementia Rating
Pirhonen et al. (2022)	05/20 -06/20		Online survey	Well-being
Plangger et al. (2022)	03/20–07/20	11/19–02/19	Assessments by researcher	MMSE, GDS-15, Beck-Angst-Inventar, QoL
Rohner et al. (2022)	08/20–09/20		Assessments by researcher	Loneliness, social isolation
Savage et al. (2022)	03/20–09/20		Public administrative data	Non-COVID-19 mortality
Schweighart et al. (2021)	12/20		Interviews	Well-being
Shum et al. (2020)	01/20–05/20	01/19–05/19	Medical records	Admission to acute medical ward due to poor oral intake
Sizoo et al. (2020)	04/20–05/20		Online survey	Well-being
Sizoo et al. (2022)	05/20–08/20	02/20–04/20	Health records and direct assessment by ECP	Neuropsychiatric symptoms, psychotropic drug use
Srifuengfung et al. (2021)	08/20–10/20		Assessments by on-site caregiver	Post-traumatic Stress Disorder Checklist (PCL-17), PHQ-9, Generalized Anxiety Disorder Scale (GAD-7)
Staempfli et al. (2022)	10/20–12/20		Interviews	Well-being
Stall et al. (2021)	03/20–09/20	04/18–02/20	Drug benefit database	Prescription of psychotropic drugs
Stevenson et al. (2022)	04/20–08/20	01/19–02/20	Retail pharmacy data	Prescription of antipsychotics, benzodiazepines, antidepressants, opioids, muscle relaxants, mood stabilizers
Sweeney et al. (2022)	06/20–12/20		Online-survey and interviews	Well-being
Tan et al. (2022)	2020	2019	Medical records	Admission to hospital for Non-COVID diseases
Thomas et al. (2022)	09/20–12/20		Interviews	Well-being
Van der Roest et al. (2020)	04/20–05/20		Online survey	Loneliness (1 item), Mental Health Inventory 5-index
Wammes et al. (2020)	04/20–05/20		Online survey	Well-being
Yan et al. (2023)	01/17–12/20		MDS	Use of antipsychotics

*ADLs*—activities of daily living, *ECP*—elderly care physicians, *GDS*—Geriatric Depression Scale, *HADS*—Hospital Anxiety and Depression Scale, *PHQ-9*—Patient Health Questionnaire-9, *RAI-MDS*—Resident Assessment Instrument-Minimum Data Set

### Characteristics of included studies

The review encompassed a total of 62 observational studies conducted in 21 different countries, which came from North America (n = 24 studies), Europe (n = 30 studies), Asia (n = 6 studies), and Oceania (n = 2 studies). Across all studies, the most common source was routine data, i.e., residents’ health records, care plans, medical claims data, or drug dispensing data (n = 20 studies). Interviews conducted by video call, telephone, face-to-face or focus groups were the next most commonly described method (n = 16 studies), involving residents (n = 9 studies), proxies such as family members, friends, and / or legal guardians of residents (n = 9 studies), and HCP (n = 4 studies). Surveys (n = 15 studies) were administered online (n = 11 studies) or as paper–pencil surveys (n = 4 studies). While two online surveys included residents, the majority of online surveys included proxies and HCP. All paper–pencil surveys included residents. Studies using data from direct assessment of residents (n = 16 studies) were conducted by on-site HCP or research staff, or used self-assessment instruments. Two studies analyzed publicly available mortality data.

The existing literature covers the range of conceptually important physical and mental health outcomes quite well (Additional file 1: Table S2). In the following, we organize further outcomes and their respective findings extracted in accordance with the conceptual background as outlined above.

### Physical and mental health consequences in light of the stress and learned helplessness conceptual perspective

#### Depressive symptoms

Prevalent feelings of anxiety, loneliness, sadness, and depressive symptoms were commonly reported in 23 studies. The sources of data were diverse. Qualitative studies including HCP and / or proxies reported an increase in depressive symptoms. One study using data from a national survey mailed to residents found an association between intensity of isolation and feelings of loneliness (Hua and Thomas 2021). Most studies of the prevalence of depressive symptoms reported increased levels compared with pre-pandemic assessments (n = 12 studies) or community-dwelling older adults (n = 2 studies) while one of these studies reported conflicting results with no significant increase in depressive symptoms compared to the three proceeding years (McArthur et al. 2021). Importantly, studies analyzing data collected at different times during the pandemic showed that depressive symptoms fluctuated with a high prevalence after the onset of social isolation and a subsequent decrease when visiting restrictions were relaxed (Angevaare et al. 2022; Górski et al. 2022a, b; Górski, Garbicz, et al., 2022; Levere et al. 2021; Plangger et al. 2022). Interviews with residents conducted during later phases of the pandemic confirm little psychological impact at that time (Schweighart et al. 2021; Thomas et al. 2022).

#### Cognitive status

Changes in cognition were explored in 17 studies. In quantitative studies using interviews and surveys, concerns about accelerated cognitive decline were expressed by residents, proxies and HCP. Using longitudinal assessments one study reported a decline in MMSE scores between October 2019 and July 2020 (Greco et al. 2021) and two others reported an increase in cognitive impairment from pre-isolation to post-isolation assessments (Górski et al. 2022a, b; Plangger et al. 2022). Routine data from the Minimal Data Set showed a decline in cognitive function during the first months of the pandemic peaking in mid-April and then improving (Levere et al. 2021). A study from Spain could not confirm accelerated cognitive decline when analyzing the trend over at least three pre-pandemic measurements and those measurements executed during the pandemic (Pereiro et al. 2021). Residents, HCP, and proxies reported decline of physical function in interviews and surveys (n = 7 studies).

#### Functional status

A decline in functional scores was confirmed in routine data from Spain (Pereiro et al. 2021) and in a study from Spain using assessment (Cortés Zamora et al. 2022). In contrast, a lack of change in physical functional status was confirmed in studies using routine data (n = 5 studies), direct assessment (Greco et al. 2021), and interviews and surveys (Kiyoshi-Teo et al. 2022).

#### Mortality

Non-COVID-19 related mortality of residents during the pandemic has been reported in studies from North America using routine data. Compared to pre-pandemic controls, mortality was not elevated in non-infected residents (Akhtar-Danesh et al. 2022) nor in residents living in LTCFs without known cases of COVID-19 infections (Barnett et al. 2022). The association between intensity of state restrictions and non-COVID-19 mortality in residents remained unclear in one study (Li et al. 2022) while another study found excess mortality in residents without family contact compared to residents with family contact (Savage et al. 2022). Lower numbers of admissions to hospital were confirmed by studies from Canada and Singapore (Jones et al. 2022; Tan et al. 2022).

### Physical and mental health Consequences in light of the social contact loss conceptual perspective

#### Loneliness

Loneliness was reported in 17 studies with information coming primarily from residents (n = 13 studies) but also from proxies (n = 8 studies) and HCP (n = 4 studies) collected from a variety of data sources. In all studies, residents, proxies and staff members reported a high prevalence of loneliness among residents. However, one study comparing results from the Swedish annual Elderly Care Survey in 2020 with a historical control could not confirm significantly higher levels during the pandemic after controlling for differences in health status (Gustafsson, Schröders, et al., 2022).

#### Quality of life

All but one of the studies addressing this aspect (n = 10 studies) used qualitative methods. The lack of stimulation and absence of care provided by visitors was expressed by residents in one study (Staempfli et al. 2022). However, concerns were not uniformly expressed, with 62% of proxies expressing concerns in a survey from Finland conducted in the summer of 2020 (Pirhonen et al. 2022). Estimates regarding cognitively impaired residents diverged, with Elderly Care Physicians (ECP) highlighting the difficulties these residents face when using video calls (Sizoo et al. 2020), while proxies in two studies estimated the impact on the quality of life of cognitively impaired residents to be less severe (Paananen et al. 2021; Wammes et al. 2020). In December 2020, residents of a German LTCF found that visiting restrictions had little impact on their quality of life, while they complained about a lack of activities and boredom (Schweighart et al. 2021).

#### Loss of appetite and body weight

Four studies using routine data early in the pandemic found substantial weight loss in non-infected residents that exceeded weight loss in previous years or pre-pandemic months. All of these studies were conducted in North America while another Dutch study using routine data could not confirm such findings when comparing changes in body weight with a pre-pandemic control group (Angevaare et al. 2022). The only study from Asia reported an increase in the number of residents with severe dementia admitted to an acute hospital between January and May 2020 due to poor oral intake (Shum et al. 2020). In interviews and surveys, HCP and proxies reported a decrease in both appetite and oral intake alike (n = 5 studies).

### Physical and mental health consequences in light of the ‘Total Institution’ conceptual perspective

#### Loss of autonomy

In one case study, a resident expressed concerns about her recovery due to the perceived loss of autonomy (Davies-Abbott et al. 2021). Feelings of reduced autonomy were attributed to factors such as lack of information, infantilization, and exclusion from decision-making, as reported by residents in another study (Kaelen et al. 2021).

#### Neuropsychiatric symptoms

In surveys, concerns about an increase in neuropsychiatric symptoms were expressed by proxies and staff members (n = 2 studies). A study from France found an increase in hallucinations during the lockdown in residents diagnosed with Alzheimer’s disease (El Haj et al. 2021a, b). Similarly, ECP from the Netherlands reported an increase in neuropsychiatric symptoms including agitation and aggression, but also increased calm in some residents living in psychogeriatric wards (Sizoo et al. 2020). Other qualitative studies have confirmed differences in responses between residents. For example, in another Dutch study, HCP reported on an increase in cohesion and social connectedness, less attention-seeking behavior, and less aggression in some residents (Leontjevas et al. 2021). In contrast, a Canadian study using routine data found no significant effect on behavioral problems and no increased prevalence of delirium (McArthur et al. 2021).

#### Use of psychoactive medications

Six studies of psychotropic medications in the United States and Canada used retail pharmacy data, drug benefit databases, and care plans. Four studies estimated a small increase in the use of psychoactive drugs. A survey of LTCF directors in Italy found increased use of benzodiazepines and antipsychotics early in the pandemic (Lombardo et al. 2020). Between March and December 2020, prescription and use of psychoactive drugs increased and then decreased. In contrast, two studies found little or no increase. Two studies from the Netherlands suggested that the overall use was unchanged while some residents received more psychoactive drugs (Sizoo et al. 2020, 2022).

## Discussion

The premise underlying this review paper was that the level of research focus on older adults in LTCFs during the COVID-19 pandemic may have been relatively less extensive compared to the overall research conducted on older adults, has mostly considered community-dwelling individuals (Resnick et al. 2021). Nonetheless, we were able to extract and analyze 62 studies on the physical and mental health consequences of contact restrictions for older LTCF residents published through November 2022. An important feature of the present review is its reliance on established theoretical perspectives with particular relevance for older adults in LTCFs, namely (1) stress and learned helplessness; (2) social contact loss; and (3) ‘total institution'.

To begin with, it is noteworthy that all of the conceptually derived outcome domains were addressed in the 62 studies, albeit with varying levels of research attention. In terms of outcomes driven by the stress and learned helplessness conceptual perspective, 23 studies focused on depressive symptoms, which was also the largest cluster able to speak to a defined outcome domain with a total of 62 studies. Firstly, 12 studies were able to consider contrasts with pre-pandemic data, and all but one found an increase during the pandemic. Second, however, such an increase in the prevalence of depressive symptoms was mostly found in studies that focused on the first months of the pandemic, whereas effects were largely leveled off in studies that focused on later pandemic phases. The level of depressive symptoms in LTCF (approximately 15 to 20 percent; McCusker et al. 2014) has been found to be about twice the level of depression in the general elderly population. The pandemic-induced increase in social contact loss, which was unexpected, threatening, and particularly challenging to comprehend for individuals with dementia-related disorders, likely amplified reactive depressive episodes. This is especially concerning as this population already experiences lower levels of social connectedness compared to the general elderly population. On the other hand, the easing of social isolation supports the transient nature of the observed increase in depressive symptoms. Crucially, the reports of professionals and relatives in a number of included studies need to be considered cautiously against the background of LTCF residents’ unmet needs and fears during the pandemic (Mitchell et al. 2021).

Findings in other areas relevant for the stress and learned helplessness perspective are less straightforward. Evidence from studies in North America has not confirmed increased mortality among non-infected residents. However, the lack of data from other parts of the world leaves this question open. These findings are supported by the lack of decline in functional ability found in many studies. While accelerated cognitive decline has been reported by HCP and proxies, scores on the MMSE and caregiver-rated cognition suggest that the observed decline may reflect the natural course of the underlying pathologies, similar to previous years. However, analyses of routine data show a fluctuating trajectory of change during the early months of the pandemic, suggesting an impact on cognitive performance nonetheless.

Adverse outcomes driven by the loss of social contact conceptual perspective, particularly loneliness, were probably the most discussed as ‘obvious’ for LTCF residents in public discourse and the media during the early stages of the pandemic. The dramatic disruption of social connections to the outside and inside social world of the LTCF was very evident. However, although a considerable number of studies addressed loneliness, findings in this area remained inconclusive. A major reason for this was the lack of comparative data that could represent pre-pandemic conditions in the study designs used. The qualitative nature of many studies in this area did not allow quantification for comparison with the pre-pandemic situation. Findings in other areas, such as quality of life, which are framed within the theory of loss of social contact, remained limited, perhaps because of the already reduced quality of life in LTCF. Although inconsistent, a limited number of studies support that appetite and weight loss seemed to be a problem during the early pandemic phases.

Adverse outcomes driven by the ‘total Institution’ conceptual perspective find some evidence in the existing study pool, but overall, conflicting findings and a rather low number of studies suggest qualifying this area as the one with lowest clearness in findings. In fact, only two studies showed frustration over resulting loss of autonomy and lack of participation in decision making. Increases in prescription of psychoactive medication remained inconclusive.

### Limitations

This review presents the results of a comprehensive search, which allowed us to obtain an overall picture of the topic. Nevertheless, there are limitations that need to be considered. First, the data presented in this review are from the first few months of the pandemic (up to December 2020). Supported by previous research on critical events, stress and coping, adaptation efforts and habituation may have mitigated the negative consequences of contact restrictions later in the pandemic (Aldwin et al. 2021). Second, there are international differences in the terminology used to describe 'care settings'. Thus, a care setting in one study may not be identical to what is called a 'long-term care facility' or 'nursing home' in another country. However, the authors were sensitive to such differences and applied consistent criteria for study inclusion. Third, this review does not critically assess the quality of the included studies due to their high heterogeneity and quantitative–qualitative designs.

## Conclusions

Public discourses about the health outcomes of LTCF residents during the COVID-19 pandemic often relied on 'face validity.' Our scoping review supports that such face validity is only partially justified. In summary, the strongest evidence suggests a temporary increase in depressive symptoms, while findings for other potential adverse outcomes were mixed. In future reviews, a thorough evaluation of study quality, along with greater research on long-term outcomes, may lead to more definitive conclusions. Given the current state of knowledge, we refrain from making concrete recommendations. However, considering our understanding of risk factors for LTCF residents beyond the pandemic and the partial evidence of potential adverse effects across different domains, it is advisable to prioritize efforts that enhance social connectivity for LTCF residents both within and outside the facility. This can include utilizing digital information and communication technologies to a greater extent.

## Registration of Protocol (2021/08/31)

At Center for Open Science (ofs.io) https://osf.io/g2yav/?view_only=38f0c6caebec4afb90378dc6726fc1ea

### Supplementary Information

Below is the link to the electronic supplementary material.

**Additional file 1**. Supplementary tables.
