# Supplementary material for: Consequences of contact restrictions for long-term care residents during the first months of COVID-19 pandemic: a scoping review
Source: Eur J Ageing. 2023 Oct 17;20(1):39. doi: 10.1007/s10433-023-00787-6 (PMC10581973; doi:10.1007/s10433-023-00787-6)
Supplement: Supplementary file 1 — Additional file 1. Supplementary tables. [file 10433_2023_787_MOESM1_ESM.pdf]

Consequences of Contact Restrictions for Long-term Care Residents During the first Months of COVID-19 pandemic – a ccoping review

European Journal of Ageing

Benzinger, Petra, Wahl, Hans-Werner, Bauer, Jürgen M., Keilhauer, Anne, Dutzi, Ilona, Maier, Simone, Hölzer, Natalie, Achterberg, Wilco M., Denninger, Natascha-Elisabeth

Corresponding author: Petra Benzinger, Email: petra.benzinger@agaplesion.de

ADDITIONAL MATERIAL

Table S1 Preferred Reporting Items for Systematic reviews and Meta-Analyses extension for Scoping Reviews (PRISMA-ScR) Checklist

SECTION	ITEM	PRISMA-ScR CHECKLIST ITEM	REPORTED ON PAGE #
**TITLE**			
Title	1	Identify the report as a scoping review.	1
**ABSTRACT**			
Structured summary	2	Provide a structured summary that includes (as applicable): background, objectives, eligibility criteria, sources of evidence, charting methods, results, and conclusions that relate to the review questions and objectives.	2
**INTRODUCTION**			
Rationale	3	Describe the rationale for the review in the context of what is already known. Explain why the review questions/objectives lend themselves to a scoping review approach.	3-5
Objectives	4	Provide an explicit statement of the questions and objectives being addressed with reference to their key elements (e.g., population or participants, concepts, and context) or other relevant key elements used to conceptualize the review questions and/or objectives.	5
**METHODS**			
Protocol and registration	5	Indicate whether a review protocol exists; state if and where it can be accessed (e.g., a Web address); and if available, provide registration information, including the registration number.	6
Eligibility criteria	6	Specify characteristics of the sources of evidence used as eligibility criteria (e.g., years considered, language, and publication status), and provide a rationale.	7-8
Information sources*	7	Describe all information sources in the search (e.g., databases with dates of coverage and contact with authors to identify additional sources), as well as the date the most recent search was executed.	6-7
Search	8	Present the full electronic search strategy for at least 1 database, including any limits used, such that it could be repeated.	Supplement S3
Selection of sources of evidence†	9	State the process for selecting sources of evidence (i.e., screening and eligibility) included in the scoping review.	7

SECTION	ITEM	PRISMA-ScR CHECKLIST ITEM	REPORTED ON PAGE #
Data charting process‡	10	Describe the methods of charting data from the included sources of evidence (e.g., calibrated forms or forms that have been tested by the team before their use, and whether data charting was done independently or in duplicate) and any processes for obtaining and confirming data from investigators.	8
Data items	11	List and define all variables for which data were sought and any assumptions and simplifications made.	-
Critical appraisal of individual sources of evidence§	12	If done, provide a rationale for conducting a critical appraisal of included sources of evidence; describe the methods used and how this information was used in any data synthesis (if appropriate).	-
Synthesis of results	13	Describe the methods of handling and summarizing the data that were charted.	8
**RESULTS**			
Selection of sources of evidence	14	Give numbers of sources of evidence screened, assessed for eligibility, and included in the review, with reasons for exclusions at each stage, ideally using a flow diagram.	27
Characteristics of sources of evidence	15	For each source of evidence, present characteristics for which data were charted and provide the citations.	26-27
Critical appraisal within sources of evidence	16	If done, present data on critical appraisal of included sources of evidence (see item 12).	-
Results of individual sources of evidence	17	For each included source of evidence, present the relevant data that were charted that relate to the review questions and objectives.	Supplement S2
Synthesis of results	18	Summarize and/or present the charting results as they relate to the review questions and objectives.	8-12
**DISCUSSION**			
Summary of evidence	19	Summarize the main results (including an overview of concepts, themes, and types of evidence available), link to the review questions and objectives, and consider the relevance to key groups.	12-14
Limitations	20	Discuss the limitations of the scoping review process.	15
Conclusions	21	Provide a general interpretation of the results with respect to the review questions and objectives, as well as potential implications and/or next steps.	15-16
**FUNDING**			
Funding	22	Describe sources of funding for the included sources of evidence, as well as sources of funding for the scoping review. Describe the role of the funders of the scoping review.	8

From: Tricco AC, Lillie E, Zarin W, O'Brien KK, Colquhoun H, Levac D, et al. PRISMA Extension for Scoping Reviews (PRISMAScR): Checklist and Explanation. Ann Intern Med. 2018;169:467–473. doi: 10.7326/M18-0850.

Table S2: Findings from included studies

First author	Context[b]	Setting	Inclusion criteria (IC)/ Exclusion criteria (EC)	Sample size (n) & characteristics of participants	Main findings related to the research questions	Outcomes
(Akhtar-Danesh et al., 2022)	CA[a]: Social outdoor visits encouraged; from 09/20, FM providing essential caregiving activities could enter the homes; as cases increase again, some areas limited social visits but enable FM to continue to see their relatives.	LTCFs	/	In 2020: n = 89,034 LTC-R	Mortality rates for LTC-R with COVID-19 negative tests were at the same level as in 2019.	General health & mortality
(Ali et al., 2022)	US[a]: Social outdoor visits encouraged; from 09/20, FM providing essential caregiving activities could enter the homes; as cases increase again, some areas limited social visits but enable FM to continue to see their relatives.	NHs	/	In 2020: n = 89,034 LTC-R	Compared to 2019, more prescriptions of antipsychotics during Q1 2020 (7.41%) and Q2 2020 (0.92%) with decline during Q3 2020, reaching 2019 levels by the end of 2020; refill prescriptions higher throughout 2020, new prescriptions increased only during Q1 2020, then decline compared to 2019.	Prescriptions of psychoactive drugs
(Angevaare et al., 2022)	NL[a]: NH were put under quarantine banning visitors; restrictions for LTC-R not to go outside and not to participate in numerous activities.	42 LTCFs	Cohort study: IC: ≥ 60 years; EC: previous assessment not on same ward, residing on ward without 24 h service<br>Focus groups: IC: direct work with LTC-R during the lockdown	Cohort study: n = 298 LTC-R (lockdown) / n = 625 LTC-R (control)<br>Focus groups: n = 15 HCP	N. s. weight change.<br>Increase in self-reported mood symptoms greater between 2 measurements in the lockdown group than in the control group, increase sign. greater only for LTC-R with assessments during the 1st half of the lockdown; observer-rated mood symptoms n.s. different; post hoc analyses with input from focus groups could not confirm findings expressed by focus group members.<br>Overall no difference but in LTC-R with no/mild CI, increase of withdrawal between two measurements lower in the lockdown group compared to the pre-pandemic control group; for LTC-R aged ≥ 90 years, increase in aggressive behavior lower in the lockdown group.	Weigth & appetite<br>Mood<br>Neuropsychatric symptoms
(Arpacıoğlu et al., 2021)	TR[a]: As of March 2020, restriction all visitors were implemented, canceling all group activities and communal dining.	Two NHs	IC: Randomly selected NH, community-dwelling older adults living in the same street; EC: CI, severe medical condition	n = 66 LTC-R, n = 67 community-dwelling adults; 67% aged 65 - 79 years, 33% ≥ 80 years, female 58.6%	Compared to community-dwellers, LTC-R had higher levels of anxiety and death anxiety, depression and lower levels of satisfaction with life.	Mood
(Barnett et al., 2022)	US[a]: From 03/20 to 11/21, federal and state governments imposed measures on SNFs, including closing their premises to all visitors and eliminating communal dining & other group activities.	SNFs	IC: Medicare beneficiaries EC: short-stay patients for temporary post-acute care	In 2020: n = 997,418 LTC-R<br>Median age 81 years, female 66.2%, 76% White non-Hispanic<br>SNF according to infection status: 41.4% active COVID-19 months, 52.4% no-known COVID-19 months, 6.2% former COVID-19 months	Among no-known COVID-19 LTCF, mortality decreased by 0.15%, hospitalizations decreased by 0.83%, and ED visits by 0.79%.<br>By 11/20, LTC-R in no-known COVID-19 LTCF had llost 1.8 lb more weight, had n. s. change in the number of ADLs requiring assistance, have worsened depressive symptoms (PHQ-9 scores began to worsen prior to 2020, suggesting pre-pandemic trends) compared with 2018 and 2019.	General health & mortality<br>Weigth & appetite<br>Cognitve & functional status<br>Mood
(Campitelli et al., 2021)	CA[a]: Visitors were restricted, group activities stopped; recreation staff redeployed to keep family connected with LTC-R; all facilities received one iPad per ten LTC-R; mid-May, in-person outdoor visits were introduced[b].	NHs	EC: concurrent inpatient admission, lack of previous MDS, missing age and/or sex, aged ≥110 years	In 2020, n = 76,549 LTC-R. Mean age 83.4 years; female 68.9%	Compared to time trend (2017 – 2019), increased use of antipsychotics, antidepressants, trazodone, benzodiazepine, opioids and anticonvulsants; absolute difference in observed vs projected use highest for antipsychotics (1.52%), followed by antidepressants (1.43%), trazodone (1.06%), opioids (1.06%), benzodiazepines (0.64%), and anticonvulsants (0.48%).	Prescriptions of psychoactive drugs
(Chang et al., 2021)	CN[a]: Visiting restrictions were implemented on 01/24/20.	Four NHs	IC: ≥ 65 years, able to participate in active recreational activities, absence of cold symptoms	n = 235 LTC-R. Mean age 77.72 years, female 57.4%	High levels of self-rated health, leisure support and flow predicted lower levels of loneliness, duration of residency in the NH associated with loneliness.	Mood

(Cheung et al., 2022)	NZ[a]: Variation in restrictions ranging from visits following a set of hygiene rules to visits only possible for designated visitors with a limit on the number of visitors, length of visit and places where visits took place.	Aged residen tial care	/	In 2020: n = Maori 538, pacific people n = 276, New Zealands Europeans n = 11,322 LTC-R	No difference for measures of cognition, ADLs, and falls between the first wave of COVID-19 and the comparative period in the three ethnic groups.<br>In 2020, fewer Māori LTC-R reported feeling lonely, New Zealand European LTC-R reported more severe depressive symptoms than during the comparative period in 2019.	Cognitve & functional status<br>Mood
(Cornally et al., 2022)	IE[a]: On 27 March, guidance on cocooning to protect people > 70 years; NH providers instructed to 'carefully discuss this advice with the families, carers and specialist doctors caring for such persons to ensure this guidance is strictly adhered to'; however, what 'cocooning' means in practice not covered in this guidance; as of 5 May, LTC-R could go outside.	LTCFs	/	n = 120 FM, friends, and legal guardians<br>69% aged 45 to 64 years, 81% visiting at least once a week, female 83%, 95% FM	Respondents perceived negative impact on general health status of LTC-R.<br>Respondents observed what they believed to be deterioration in activities of daily living; perceived negative impact on cognition of LTC-R.	General health & mortality<br>Cognitive & functional status
(Cortés Zamora et al., 2022)	ES[a]: On March 7, all LTCF were closed to external visitors, LTC-R were isolated.	Five NHs	IC: ≥ 65 years; EC: Global Deterioration Scale ≥5, poor general condition, aphasia, blindness, hospitalization, inability to contact a legal representative	n = 215 LTC-R	N.s. change in ambulation between baseline visit and three-month follow-up; functional loss median 5 points in BI.	Cognitive & functional status
(Danilovich et al., 2020)	US[a]: All non-essential visitors and group activities were ceased and meals delivered in-room; residents were encouraged to stay in their rooms.	Single NH	IC: min. of one weight measurement per month from 12/19 through 04/20	n = 166 LTC-R. Median age 86.9 years (range 61–102 years), female 67.5%, 60.8% CI, 52.2% diagnosis of depression or anxiety	LTC-R weighed 3.68 lbs less after implementing restrictions than they averaged in the 3 months before restrictions, controlled for confounders.	Weigth & appetite
(Davies-Abbott et al., 2021)	US[a]: In March, main care home chains stopped non-essential visits although no formal ban on visits to NH was issued; guidance on family visits issued on the July 22, advising that visits were limited to a 'single constant visitor.	Single NH	IC: diagnosis of dementia	n = 1<br>71 year old female diagnosed with fronto-temporal dementia	This LTC-R still able to find ways to promote her autonomy and decision-making; five themes reveal how the participant responded to the pandemic: autonomy, fears, keeping connected, keeping safe and other people living with dementia.	Quality of life
(Egeljić-Mihailović et al., 2022)	RS[a]: Limitations of movements and social contacts on adults aged >65; LTC-R spent most of their time in total isolation.	Two NHs	IC: > 60 years, MMSE > 24; EC: poor health, disorientation, impaired vision or hearing, speech and language disorders, psychiatric illness, malignant diseases.	n = 100 LTC-R. Mean age 79.22 years, female 59.1%;<br>n = 189 community-dwelling older adults. Mean age 70.9 years, female 60.3%	GDS higher (7.54) in LTC-R vs. community-dwellers (6.07); social participation higher (0.71) in community-dwellers vs. LTC-R (1.21).	Mood
(El Haj et al., 2020)	FR[a]: Almost all visits stopped and access to non-essential, communal activities, and non-essential personnel restricted; in 05/20, visits behind plexiglass or behind windows possible.	Retire-ment homes[d]	IC: probable AD, MMSE ≥21 (assessment within 3 months prior the first wave of the pandemic)	n = 58 LTC-R. Mean age 71.8 years, female 63.8%	Higher scores for depression and anxiety during the pandemic as compared to before the pandemic.	Mood
(El Haj, Larøi, et al., 2021)			IC: Clinical diagnosis of probable AD with hallucinations before lockdown; participants drawn from an ongoing study	N = 47 LTC-R.Mean age 71.9 years, female 57.5%, mean MMSE 14.3	Increased hallucinatory experiences during the lockdown compared to before the pandemic.	Neuropsychiatric symptoms
(El Haj, Moustafa, et al., 2021)			IC: Clinical diagnosis of probable AD	n = 72 LTC-R. Mean age 72.9 years, female 59.7%, mean MMSE 19	Higher scores on depressive symptoms during the lockdown as compared to before the pandemic.	Mood

(El Haj et al., 2022)	FR[a]: 1st lockdown implemented 03/16/20 - 05/11/20, followed by an easing of restrictions; on 10/30/20, 2nd lockdown imposed and partially alleviated 11/27/20; during 2nd lockdown visits allowed under strict conditions, activities and services considered as non-essential restricted.		IC: diagnosis of probable AD	n = 62 LTC-R. Mean age 72.17 years, female 64.5%, mean MMSE 19.89	Higher levels of depression, anxiety and loneliness during the 2nd lockdown than during the 1st lockdown.	Mood
(El Haj & Gallouj, 2022)	FR[a]: Retirement homes in France restricted visits in 03/20. All activities considered as non-essential suspended, including restricting access to non-essential personnel, group activities, and communal dining.		IC: probable AD; EC: MMSE < 21	n = 63 LTC-R. Mean age 70,13 years, female 61.9%, mean MMST 22.01	High level of loneliness	Mood
(Gerlach et al., 2021)	NL/UK[a]: NH were advised to restrict visitation of all visitors and non-essential HCP, and to cancel communal dining and all group activities.	440 NHs	IC: > 64 years, diagnosis of dementia;<br>EC: schizophrenia, Tourette's, or Huntington's disease	n = 14,025 LTC-R. Mean age 85 years, white 82%, female 72%, Medicaid eligible 89%, diagnosis of anxiety 39%, diagnosis of depression 53%	No changes in prevalence of antipsychotic, antianxiety and hypnotic use, small sign. increase in use of anti-depressants (54.8% to 57.2%) and opioids (23.9% to 25.6%).	Prescriptions of psychoactive drugs
(Giebel et al., 2022)	NL/UK[a]: At the point of data collection, different restrictions in place for each NH; in the NL, all LCF-R were scheduled to receive a visitor once during the week.	NHs	IC: FM > 18 years	n = 125 FM (n = 99 from NL, n = 26 from UK)<br>UK: female 69,2%<br>NL: female 50 %	Concerns over deterioration of health and well-being of LTC-R.	General health & mortality
(Górski, Buczkowska, et al., 2022)	PO[a]: In many facilities, limitation of or ban on visits, restricting contacts between LTC-R, sometimes between LTC-R and staff, many institutions ceased group activities and communal meals.	NHs	IC: residency for > 30 d, maintenance for basic ADLs; EC: short-stay, severe illness with prolonged bed rest, depression, acute positive symptoms of schizophrenia	n = 273 LTC-R. Mean age 80.8 years, female 51.6% , 32% had a duration of stay at NH > 24 months	Increase in CI from 03/20 to 12/20: 84% to 90% for any cognitive impairment, 28% to 31% for severe dementia, respectively<br>In 02/20, no LTC-R at risk of severe depression and 14% at moderate risk of depression; in 12/20, 2.6% at risk of severe depression and 45.4% at moderate risk of depression, respectivelly. No gender difference.	Cognitive & functional status<br>Mood
(Górski, Garbicz, et al., 2022)		Single NH	IC: residency from at least 02/20 to 05/20, able to complete GDS	n = 58 LTC-R. Mean age 84.12 years, female 70.7%, normal MMSE score in 10.4%, severe dementia in 24.1%	Immediately after beginning of the lockdown 72.4% of LTC-R did not suffer from depressive symptoms as compared to 87.9% in 02/20; after relaxation of measures in 05/20, number of individuals with no depressive symptoms increased; mean GDS score increased from pre-pandemic level during lockdown 03/20, and declined in 04/20 and further declined in 05/20.	Mood
(Greco et al., 2021)	IT[a]: NHs were advised to restrict visitation of all visitors and non-essential HCP and to cancel communal dining and all group activities.	Single NH	Inclusion in ongoing study between 10/19 to 12/19 alive in 03/20	76 LTC-R infected with SARS-CoV-2, 20 LTC-R deceased, 76 matched controls<br>Baseline: Cases / controls: mean age 84.4 years / 85.1 years, female 16% / 17%, mean MMSE 16.3 / 16.8, respectively	10% decrease in MMSE scores in cases and in controls; n.s. change in walking speed, hand grip strength, and frailty prevalence between 10/19 and 07/20 in non-infected LTC-R.	Cognitive & functional status
(Gustafsson, Fonseca-Rodrígue z, et al., 2022)	SE[a]: Initially, advise against visiting NH; subsequently a ban was introduced (from April 1); LTC-R were allowed to leave the NH any time.	NHs	IC: ≥ 65 years, recipients of home care or residence at LTCF	In 2020: n = 23,536 LTC-R, n = 73,386 community-dwellers<br>≥ 85 years: 56.4%, female 65.8%	In 2020, prevalence of loneliness 3.66 percentage points higher among LTC-R compared to community-dwellers as compared to a difference of 1.81 point in 2019.	Mood

(Gustafsson, Schröders, et al., 2022)			IC: respondents who completed 2019 and 2020 surveys; ≥ 70 years in 2020	n = 11,782 LTC-R<br>In 2020, mean age 88.2 years, female 71%	Small increase in loneliness prevalence from 2019 (17%) to 2020 (19%), after taking variations in health into account results n.s.	Mood
(Hindmarch et al., 2021)	CA[a]: Public health measures reduced / eliminated visiting access to assisted/supportive living and LTCF.	LTCFs	IC: FM over the age of 18 providing care for a PLWD in LTC	n = 70 FM, friends, and legal guardians of LTC-R (survey), n = 7 FM (focus group)<br>Mean age 59 years, female 76 %<br>PLWD: mean age 76 years, female 67%	PLWD showed<br>dementia progression.<br>an increase in responsive behaviours (51%)	Cognitive & functional status<br>Neuropsychiatric symptoms
(Ho et al., 2022)	CN[a]: Face-to-face visits by outsiders, including FM and volunteers, were terminated by LTCFs in Hong Kong. They also stopped holding formal group or outdoor activities.	Seven LTCFs	IC: ≥ 60 years, residents during the COVID-19 outbreak in Hong Kong (i.e., starting from 01/28/20), able to speak Cantonese, Putonghua, or English, ≥ 6 Abbreviated Mental Test	n = 15 LTC-R. Mean age 83.4 years, female 80%	Exacerbation of older adult’s disconnection with prior commitments reinforcing a pessimistic view of the future; sense of vulnerability culminated in older adults experiencing difficulties to make sense of their selves; when older adults experienced collapse in self-interpretation, their loneliness appeared more pronounced. They were unable to establish a meaningful identity resulting in feelings of self-worthlessness.	Mood
(Hovey & Shropshire, 2021)	US[a]: NH were advised to restrict visitation of all visitors and non-essential HCP, and to cancel communal dining and all group activities.	LTCFs	IC: FM of residents in LTCF, informal caregiving responsibilities, no visits due to mandated closures	n = 14 CG	LTC-R were perceived as being lonely, depressed, scared and lacking understanding of the situation.	Mood
(Hua & Thomas, 2021)	US[a]: 45.2% LTC-R lived in communities that prohibited them from leaving their rooms. 64.9% of LTC-R lived in a community that stopped providing group activities in the common area.	LTCFs	EC: surveys completed by proxies	n = 123 LTC-R	28.7% LTC-R reported feeling lonelier during the pandemic than in a typical week prior to the pandemic; more LTC-R who were not allowed to leave their rooms felt lonelier (40.7%) compared to LTC-R who were allowed to leave their rooms (18.7%); other measures nor associated with increase in loneliness.	Mood
(Ickert et al., 2021)	CA[a]: Entrance was restricted, as well as type and location of allowable visits; LTC-R not able to participate in typical recreation, dining, and social activities.	LTCFs	LTC-R<br>IC: moved in before 03/20, own decision maker, physically able to participate in interview<br>FM<br>IC: LTC-R moved in before 03/20	n = 14 LTC-R, female 50%<br>n = 18 FM, female 89%	Most LTC-R stated that they had not experienced a decline in their physical health. Three LTC-R noted improvements in their physical health during the pandemic<br>Several FM noted declines in the mental health; some FM observed that it was difficult to distinguish between deterioration resulting from the COVID-19 restrictions, and from the progression of dementia.<br>LTC-R discussion of wellbeing predominately focused on their psychosocial wellbeing: sadness, loneliness, fear, and frustration; not all LTC-R described deterioration in their wellbeing.	General health & mortality<br>Cognitive & functional status<br>Mood
(Jones et al., 2022)	CA[a]: Social outdoor visits encouraged; from 09/20, FM providing essential caregiving activities could enter the homes; as cases increase again, some areas limited social visits but enable FM to continue to see their relatives.	LTCFs	IC: admission to general internal medicine wards for non-COVID-19 reasons; EC: admission for COVID-19	Pre-pandemic: median age 84 years, female 47%, 38% dementia diagnosis; pandemic: median age 83 years, female 51%, 38% dementia diagnosis	Monthly admission count lower in 2020 than the average of the previous two years; relative difference greatest in 04/20; lower rate of admissions for pneumonia and higher rates for delirium with comorbidity profiles similar; rates of ICU admission similar; pandemic LTC-R more likely to die in hospital.	General health & mortality
(Kaelen et al., 2021)	BE[a]: Visitors banned and restrictions imposed e.g. cessation of social and physical activities, all meals served in the rooms; LTC-R not allowed to leave facilities.	Eight NHs	LTC-R: MMSE 24-30<br>HCP: various professions, in direct contact with residents	n = 56 LTC-R, n = 44 HCP<br>LTC-R: median age 85 years (58 to 101 years), female 62.5%<br>HCP: nurses 22.7%, nursing aids 38.6%, therapists 20.5%, support staff 18.2%	LTC-R reported<br>loss of appetite and<br>cognitive and physical deterioration with increased tiredness, loss of mobility.<br>loss of autonomy and feeling of being abandoned with wellbeing severely impacted. With ongoing restrictions, residents became	Weigth & appetite<br>Cognitive & functional status<br>Quality of life

					increasingly angry and stressed, feeling sad, losing interest, having a low morale.	
(Kiyoshi-Teo et al., 2022)	US[a]: Measures mandated LTC-R to confine to their rooms, pause their social activities, including dining and other gatherings, and maintain strict visitation restrictions.	Two ALFs	IC: participation in parent study: high fall risk, MoCA ≥15; still residing in ALF and well-enough to participate	n = 13 LTC-R. Mean age 87.1, female 85%, mean MoCA score 21.77	Negligible decrease of belief in the importance of fall prevention, small decrease in their confidence to prevent falling during COVID. Several participants emphasized that their fall risks are not different because of the pandemic; some described how the pandemic was protective for their fall risks; some reported their activity level went down, others maintained their physical activities.<br>Many participants were experiencing loneliness due to lack of social connection with important others such as family, friends, and neighbor.	Cognitve & functional status<br>Mood
(Koopmans et al., 2022)	US[a]: Study period = first 3 weeks of reopening the doors of Dutch NH starting in 05/11/20.	26 pilot NHs	/	Questionnaire: n = 46 HCP<br>Interviews: n = 73 visitors, n = 72 HCP	HCP observed an increase in the loneliness during the lockdown and a decrease in the residents'well-being.	Mood<br>Quality of life
(Leontjevas et al., 2021)	NL[a]: NH were put under quarantine banning visitors; restrictions for LTC-R not to go outside and not to participate in numerous activities.	NHs	IC: NH psychologists, elderly care physicians, nurse practitioners	n = 323 (survey) / n = 16 (interviews)<br>Survey: 62% psychologists, 24% ECP, 10% nurse practitioners, 4% other professionals;<br>Units: 55% dementia special care, 24% closed units<br>Interviews: psychologists (n = 11), ECP (n = 3), nurse practitioner (n = 1), senior manager (n = 1)	Decrease in appetite reported by staff.<br>Decrease in physical and cognitive functioning.<br>Increase of elevated mood, decrease in agitation, aggression, sleeping problems, and attention-seeking behavior; increase in cohesion and social connectedness in some LTC-R; in LTC-R with advanced dementia predominantly positive effects on neuro-psychiatric symptoms while in LTC-R without dementia predominantly negative effects; newly admitted LTC-R specifically impacted.	Weigth & appetite<br>Cognitive & functional status<br>Neuropsychiatric symptoms
(Levere et al., 2021)	US[a]: NH were advised to restrict visitation of all visitors and non-essential HCP, and to cancel communal dining and all group activities.	224 NHs	IC: living in the facility for at least 100 days prior to 03/09/20	n = 14,510 LTC-R. Mean age 80 years, female 67%, non-Hispanic white 82%	Unplanned substantial weight loss starting in mid-April, on-going. Percentage of residents with unplanned weight loss lower for non-infected LTC-R.<br>Increased prevalence of incontinence; worsened cognitive functioning, peaking in mid-April to mid-May with subsequent decline.<br>Increased prevalence of depressive symptoms peaking in mid-April to mid-May, with subsequent decline.	Weigth & appetite<br>Cognitive & functional status<br>Mood
(Li et al., 2022)	US[a]: NH were advised to restrict visitation of all visitors and non-essential HCP, and to cancel communal dining and all group activities.	LTCFs	/	n = 14,046 NH, n = 6,829 NH in states with low COVID-19 restrictions and n = 7,217 in states with high restrictions	Non-COVID-19 death rates higher in NHs in states with high compared to those in states with low COVID-19 restriction; sensitivity analyses did not suggest an association between state COVID-19 restrictions and non-COVID-19 deaths.	General health & mortality
(Lombardo et al., 2020)	IT[a]: All visits from relatives and non-essential caregivers were stopped.	NHs	IC: NH directors of a NH listed on online map of services for PLWD	n = 1,356 NH directors<br>77% of participating facilities located in the Northern Italy	33% reported at least one adverse event; 62% adopted physical restraints measures.<br>6% reported increase in the use of psychoactive drugs, mainly antipsychotics and benzodiazepine.	Neuropsychiatric symptoms<br>Prescriptions of psychoactive drugs
(Martinchek et al., 2021)	US [a]: Large outbreak of COVID-19 infections; infection control measures such as visitor restriction and dining hall closure were implemented.	Single SNF	/	n = 206 LTC-R. Mean age 75.3 years, female 56.0%, 93.3% Black, 67.9% CI; 172 infected, 32 non-infected	COVID-positive group: weight loss of 4.6% (5.8 lbs) from starting weight, COVID-negative group: weight loss of 2.4% (3.3 lbs) from starting weight	Weigth & appetite

(McArthur et al., 2021)	CA[a]: Visitors were restricted, group activities stopped; recreation staff redeployed to keep family connected with LTC-R; all facilities received one iPad per ten LTC-R; mid-May, in-person outdoor visits were introduced[c].	Seven LTCFs	/	n = 765 LTC-R<br>At first assessment: mean age 81.4 years, female 59.5 %, diagnosis of dementia 55.6%	N. s. effect on rates of delirium and depression.<br>N. s. effect on rates of behavioral problems.	Cognitive & functional status Mood, Neuropsychiatric smyptoms
(Nair et al., 2021)	MY[a]: Visitors banned.	30 NHs	/	n = 224 LTC-R. Mean age 80.50 years, female 73.2%	Prevalence of depression 94.2%, with majority of the respondents severely depressed (50.9%); most LTC-R reported anxiety level as mild (36.6%) or moderate (38.4%)	Mood
(Nash et al., 2021)	US[a]: NH were advised to restrict visitation of all visitors and non-essential HCP, and to cancel communal dining and all group activities.	RCFs	IC: self-identification as a unpaid CG for a LTC-R	n= 518 CG. Mean age 59.7, female 96,5%	Many CG reported rapid decline in their care recipient's mental and physical state as soon as the lockdown began.	Cognitive & functional status
(Paananen et al., 2021)	FI[a]: Visitors and professionals who were not part of the regular staff barred from entering; when the total lockdown eased, NH allowed some visits.	23 NHs	IC: FM of LTC-R	n = 41 FM. Age range 40 to 83 years, female 85.4%, mostly daughters, 34 / 41 LTC-R discussed had a memory disorder	Some FM reported progression of memory disorder, many reported worsening of physical functioning.<br>Many FM reported increased residents' feeling of loneliness, sadness and anxiousness. PPE caused anxiety and fear among some of the LTC-R.<br>Most of the perceived impacts were negative (n = 345/380). Few FM believed that LTC-R were not particularly affected, often attributed to memory disorders.	Cognitive & functional status<br>Mood<br>Quality of life
(Pirhonen et al., 2022)	FI[a]: In March, visits to NH was banned including HCP not part of the regular nursing staff thus effectively ending the social and leisure-time activities of LTC-R.	NHs	/	n = 366 FM. Most common age group 50-59 years, female 88 %, most FM were offspring of LTC-R.	62% reported having noticed changes in the wellbeing of their relatives.	Quality of life
(Pereiro et al., 2021)	ES[a]: Study period 2 weeks to 3 months after the relaxation of the restrictions.	Single NH	IC: residency in the care facility for the entire period of confinement (three months); ≥ 60 years; 3 measurements prior to the lockdown	n = 98 LTC-R. Mean age 83.4 years, female 62%	Decreasing trends of the cognitive and functional scores starting in pre-lockdown measurements; n. s. altered general pattern of age-related decline.<br>Depressive symptoms sign. increased in the post-lockdown measurement as compared to three pre-lockdown measurements	Cognitive & functional status<br>Mood
(Plangger et al., 2022)	AT[a]: Seclusion of NH from the residual population; access to psychosocial therapies, church services, and interpersonal contacts was restricted, group activities canceled; staff had to reduce personal interactions.	Five NHs	EC: psychiatric conditions	n = 49 LTC-R. Mean age 80.8 years, female 75.5%, CI 47%	Cognitive performance decreased sign. during the isolation phase compared to the pre-isolation phase.<br>Sign. deterioration of depressive symptoms between pre- and post-isolation assessments; sign. increase of anxiety between pre-isolation and post-isolation with consecutive decrease on follow-up.<br>Subjective QoL decreased sign. between pre-isolation and post-isolation measurements.	Cognitive & functional status<br>Mood<br>Quality of life
(Rohner et al., 2022)		16 NHs	IC: ≥ 3 months in long-term care, MMST ≥ 17, good communication skills	n = 259 LTC-R; n = 23 < 70 years; n = 59 70-79 years, n = 111 80-89 years, n = 66 ≥ 90	61% expressed feeling of loneliness, 74% felt socially integrated.	Mood
(Savage et al., 2022)	CA[a]: Social outdoor visits encouraged; from 09/20, FM providing essential caregiving activities could enter the homes; as cases increase again, some areas limited social visits but enable FM to continue to see their relatives.	LTCFs	/	n = 67,589 LTC-R	During the pandemic period, 57.8% relative increase in mortality in LTC-R without family or friend contact vs. 17.1% increase in LTC-R with family or friend contact, as compared to 01/17 – 03/20, representing 34.8% greater excess mortality; LTC-R without contact had lower hospital transfer rates prior to death in 04/20 and 05/20 despite similar rates in 03/20.	General health & mortality

(Schweighart et al., 2021)	DE [a]: Access for visitors, non-essential HCP, and volunteers restricted; communal activities ceased; during the study period, some restrictions had been eased but visits still restricted.	Two NHs	IC: diagnosis of depression as confirmed by DIA-S (Depression im Alter Skala)	n = 9 LTC-R.<br>Mean age 81.9 years, female 56%, residency in NH between one month and four years	No or only little psychological stress caused by the pandemic.<br>Not much change in emotional wellbeing, and little fear of infection.	Mood<br>Quality of life
(Shum et al., 2020)	CN: [a] Visiting restrictions were implemented on 01/24/20.	Two acute medical wards	IC: admission due to poor oral intake as judged by staff	n = 24 LTC-R<br>Admissions in 2020: mean age 89.7 years	More LTC-R with severe dementia admitted because of poor oral intake than in 2019, more cases with initiation of tube feeding.	Weigth & appetite
(Sizoo et al., 2020)	NL [a]: All visitors of LTCFwere restricted except for LTC-R in the dying phase.	/	IC: ECP in-training and their supervisors	n = 76 ECP	Decreased oral intake.<br>ECP reported physical deterioration and in psycho-geriatric units, rapid cognitive decline.<br>ECP observed loneliness, depressive symptoms, increase in somatic symptoms such as pain.<br>In psycho-geriatric units, rapid cognitive decline and changes in neuropsychiatric symptoms, increased psychotropic drug prescriptions for some but also peace for other residents.<br>QoL of most LTC-R seriously decreased; greater impact on those with dysarthria or dementia unable to understand / use video calls.	Weigth & appetite<br>Cognitive & functional status<br>Mood<br>Neuropsychiatric symptoms/ Prescriptions of psychoactive drugs<br>Quality of life
(Sizoo et al., 2022)	NL [a]: Strict visitors ban for most LTC-R until 06/20; in 08/20, visits still restricted in many facilities; limited allowance to move freely in and around the NH in 40.9% of LTC-R in 05/20, reduced to 13.6% in 08/20; almost 60% of the care practitioners not involved in direct (medical) care not allowed to visit LTC-R in 05/20, decreasing to < 4% in August.	Psycho-geriatric units at 19 NHs	IC: diagnosis of dementia, admittance to psychogeriatric unit before 01/01/20<br>EC: life expectancy < 1 month	n = 252 LTC-R. Mean age 84 years, female 71.4%; 69.8% with severe dementia	Number of LTC-R showing any NPS sign. decreased between 04/20 and 08/20; severity of depression and agitation decreased sign. during the study period; for apathy, anxiety, and psychotic behavior, n.s.. changes over time; psychotropic drug use remained largely stable over time.<br>Psychotropic drug use remained largely stable between 04/20 and 08/20.	Neuropsychiatric symptoms<br>Prescriptions of psychoactive drugs
(Srifuengfung et al., 2021)	TH [a]: In 04/20, infection control measures implemented: physical distancing, activities in open spaces with at least 2 m between each LTC-R, separate beds and personal items 1-2 m apart, refrain from going in and out of NH; outside people / organizations not permitted to arrange LTCF activities, relatives prohibited from visiting.	Two LTCFs	IC: ≥ 60 years; EC: diagnosis of dementia, Mini-Cog <3, inability to understand or communicate in Thai language, active psychosis; unwilling to sit for a 30-minute study-related interview.	n = 200 LTC-R. Mean age 76.4 years, female 66.5%	Degree of health impact rated as moderate to severe by 68.0% of LTC-R, mainly due to difficulty seeing their physicians; 37.5 stated that their medication was insufficient.<br>No or mild psychological stress reported by 70% LTC-R, post-traumatic stress by 5.5%; depressive symptoms in 7.0%, anxiety by 12%; degree of health impact rated as moderate to severe by 68.0%, mainly due to difficulty seeing their physicians. 37.5% of LTC-R stated that their medication was insufficient.	General health & mortality<br>Mood
(Staempfli et al., 2022)	CA [a]: Restriction of all visitor access in 03/20, followed in 05/20 by limited access for one “designated visitor”; exceptions in extenuating circumstances (eg, failing health); social distancing for all LTC-R.	Single LTCF	FM: IC: having a relative living in the LTCF home during the pandemic<br>LTC-R: /	n = 11 FM; female 91%,residence of relatives at the LTCF for 2-14 years<br>n = 10 LTC-R<br>One LTC-R 53 years, all others ≥65years, female 80%, 1.5–20 years of residence at the LTCF	LTC-R experienced weight loss and decrease in cognitive capacity.<br>They described feelings of loneliness, isolation, and anger.<br>Absence of care provided by visitors made sign. contributions to LTC-R's QoL; some LTC-R said that lack of access to social stimulation negatively affected their quality of life.	Weigth & appetite<br>Cognitive & functional status<br>Mood<br>Quality of life
(Stall et al., 2021)	US[a]: NH were advised to restrict visitation of all visitors and non-essential HCP, and to cancel communal dining and all group activities.	623 NHs	/	n = 77,291 LTC-R	Small absolute increases in LTC-R who received prescriptions for psychotropic drugs at the onset of the COVID-19 pandemic that persisted through 09/20, disproportionate to expected secular prescribing trends.	Prescriptions of psychoactive drugs

(Stevens on et al., 2022)		NHs & ALFs	IC: ≥ 65 years; with LTC Longitudinal Prescription Claims	01/19: n = 415,012 LTC-R	Prevalence and initiation of use of antipsychotics, benzodiazepines, antidepressants, opioids, muscle relaxants, and mood stabilizers largely unchanged; among new admissions, initiation of antipsychotic use increased by 08/20; similar trends for benzodiazepines and short-acting opioids.	Prescriptions of psychoactive drugs
(Sweeney et al., 2022)	IE [a]: On 27 March, guidance on cocooning; NH providers instructed to strictly adhered to this guidance; however, no clear definition of 'cocooning'; as of 5 May, LTC-R could go outside.	RCFs	IC: residency in the Republic of Ireland	Survey: n = 77 HCP, n = 28 FM, n = 2 LTC-R;<br>Interviews: n = 11 FM	FM described<br>- acute decline in LTC-R physical health which included reduced mobility, pressure sores, dehydration.<br>- weight loss and dehydration<br>- acute decline in LTC-R physical health which reduced mobility.<br>Staff reported loneliness, psychological / emotional needs of LTC-R not met	General health & mortality<br>Weigth & appetite<br>Cognitive & functional status<br>Mood
(Tan et al., 2022)	SG [a]: Lockdowns of NH instituted in 05/20; health-care support augmented when the first COVID-19 outbreak was detected with extra funding, manpower and resources mobilised to contain the outbreak.	Single hospital	IC: admission to hospital	2019: n = 466 admissions; 2020: n = 361 admissions of LTC-R	Number and percentage of admissions sign. decreased (7% in 2019 vs. 4% in 2020); length of stay decreased non-sign. from 21 d to 17 days; sign. decrease in pneumonia and increase in falls as a primary diagnosis on admission.	General health & mortality
(Thomas et al., 2022)	AU [a]: Mid- March, visits were restricted and banned in some areas; visits took place in LTC-Rs' rooms; later guidance that no visitors or non-essential HCP were permitted, and LTC-R should not leave the LTF.	Four RCFs	EC: LTCF experiencing an outbreak of COVID-19<br>LTC-R: CI or receiving palliative care<br>FM with a recent loss of a family member	n = 10 FM, n = 6 LTC-R, n =5 HCP	LTC-R reported few impacts on themselves; FM reported physical decline in LTC-R due to less exercise, reduced mobility, and cognitive decline / worsening dementia.<br>LTC-R reported few impacts on themselves.<br>FM reported some LTC-R to be lonely, sad, crying, annoyed, and even frightened; other observed a worsening of depression and anxiety and withdrawing.	Cognitive & functional status<br>Mood
(Van der Roest et al., 2020)	NL [a]: Visitors were banned in all LTCF; many facilities closed social facilities and stopped daytime programs.	LTCFs	/	n = 193 LTC-R, n = 1,387 FM, n = 246 HCP working in direct care<br>FM: 61% FM of LTC-R with CI HCP: 39% working in psychogeriatric units	High levels of loneliness, depression, and a sign. exacerbation in mood and behavioral problems. Residents without cognitive impairment seemed to be the most affected.<br>Sign. exacerbation in mood and behavioral problems.	Mood<br>Neuripsychiatric symptoms
(Wamme s et al., 2020)	NL [a]: Visitors banned; many facilities closed social activities.	NHs	IC: FM or friends, who had a family member or friend in a NH during the period of the visiting restrictions	n = 1,997 FM / friends. Mean age 60.1 years (range 20 - 97 years), female 74%	Concerns expressed regarding increase of<br>- cognitive impairment<br>- depressive symptoms<br>Most relatives concerned about LTC-R experiencing loss of QoL.	Cognitive & functional status<br>Mood<br>Quality of life
(Yan et al., 2023)	US [a]: NH were advised to restrict visitation of all visitors and non-essential HCP, and to cancel communal dining and all group activities.	LTCFs	IC: Medicare-eligible, reported as Hispanic, Black, or White; diagnosis of ADRD between 2017 and 2020	n = 2,787,961 LTC-R over the period of 2017-2020	Between 2017 Q1 and 2020 Q1, frequency of antipsychotic use declined from 23.7% to 23.1%; by 2020 Q4 it increased to 24.8%.	Prescriptions of psychoactive drugs

[a] Abbreviation taken from United Nations Code for Trade and Transport Locations; [b] Contact restrictions as provided by the authors or taken from https://ltccovid.org/international-living-report-covid-ltc/; [c]proportion of residents who had family contact other than in-person (e.g., e-mail, letters) unchanged (35%), proportion of residents with high social engagement within the home not different before or during lockdown; [d]number not provided;
/ : Information not provided, AD: Alzheimer Disease, ADLs: activities of daily living, ALF: assisted living facility, CG: care giver, CI: cognitive impairment, ECP: elderly care physicians, FM: family member(s), GDS: Geriatric Depression Scale, HCP: health care professionals, LTCF: long-term care facility, LTC-R: long-term care resident(s), MMSE: Mini Mental State Examination, MoCA: Montreal Cognitive Assessment, NH: nursing home(s), PLWD: person living with dementia, PPE: personal protective equipment, SNF: skilled nursing facility

Table S3 Search strategy for MEDLINE via PubMed

Search number	Search Details
28	("dutch"[Language] OR "german"[Language] OR "english"[Language]) AND 2020/01/01:2021/05/31[Date - Publication] AND #27
27	#15 AND #26
26	#16 OR #17 OR #18 OR #19 #20 OR #21 OR #22 OR #23 OR #24 OR #25
25	"covid 19"[MeSH Major Topic]
24	"CORONAVIRUS EPIDEMIC"[Text Word]
23	"CORONAVIRUS PANDEMIC"[Text Word]
22	"COVID?19"[Text Word]
21	"SARS?COV?2"[Text Word]
20	"2019?NCOV"[Text Word]
19	"2019 NOVEL CORONAVIRUS"[Text Word]
18	"CORONAVIRUS DISEASE-19"[Text Word]
17	"CORONAVIRUS DISEASE 2019"[Text Word]
16	"SARS CORONAVIRUS 2"[Text Word]
15	#1 OR #2 OR #3 OR #4 OR #5 OR #6 OR #7 OR #8 OR #9 OR #10 OR #11 OR #12 OR #13 OR #14
14	"assisted living facilities"[MeSH Major Topic]
13	"homes for the aged"[MeSH Major Topic]
12	"nursing homes"[MeSH Major Topic]
11	"assisted living facilit*"[Text Word]
10	"SKILLED NURSING"[Text Word]
9	"RESIDENTIAL FACILIT*"[Text Word]
8	"RESIDENTIAL CARE"[Text Word]
7	"RESIDENTIAL NURSING"[Text Word]
6	"AGED CAR*"[Text Word]
5	"HOMES FOR THE AGED"[Text Word] OR "HOME FOR THE AGED"[Text Word]
4	"RETIREMENT HOME*"[Text Word]
3	"LONG?TERM CARE"[Text Word]
2	"NURSING HOME*"[Text Word]
1	"CARE HOME*"[Text Word]
